# Supplementary material for: Effectiveness of ongoing single dose rifampicin post-exposure prophylaxis (SDR-PEP) implementation under routine programme conditions—An observational study in Nepal
Source: PLoS Negl Trop Dis. 2024 Dec 4;18(12):e0012446. doi: 10.1371/journal.pntd.0012446 (PMC11649142; doi:10.1371/journal.pntd.0012446)
Supplement: S1 Annex — (DOCX) [file pntd.0012446.s001.docx]

## **S1_Annex_Recorded_index_cases_and_screened_contacts_per_cohort_districts**

| **District** | **Year** | **Leprosy cases** | **Contacts screened** |
| --- | --- | --- | --- |
| Jhapa | 2015 | 188 | 8,021 |
| Morang | 2015 | 216 | 3,517 |
| Sunsari | 2015 | 121 | 3,425 |
| Udayapur | 2015 | 27 | 675 |
| Jhapa | 2016 | 157 | 4,486 |
| Morang | 2016 | 202 | 7,152 |
| Sunsari | 2016 | 116 | 3,322 |
| Udayapur | 2016 | 26 | 650 |
| Jhapa | 2017 | 139 | 3,341 |
| Morang | 2017 | 188 | 4,293 |
| Sunsari | 2017 | 96 | 2,711 |
| Udayapur | 2017 | 39 | 975 |
| Jhapa | 2018 | 276 | 3,125 |
| Morang | 2018 | 151 | 4,423 |
| Sunsari | 2018 | 84 | 2,248 |
| Udayapur | 2018 | 48 | 1,200 |
| Jhapa | 2019 | 164 | 1,278 |
| Morang | 2019 | 176 | 1,280 |
| Sunsari | 2019 | 67 | 1,634 |
| Udayapur | 2019 | 35 | 875 |
| **Total** |  | **2,516** | **58,631** |
