# Supplementary material for: Effectiveness of ongoing single dose rifampicin post-exposure prophylaxis (SDR-PEP) implementation under routine programme conditions—An observational study in Nepal
Source: PLoS Negl Trop Dis. 2024 Dec 4;18(12):e0012446. doi: 10.1371/journal.pntd.0012446 (PMC11649142; doi:10.1371/journal.pntd.0012446)
Supplement: Attachment — Submitted filename: PLOSNTD PNTD-D-24-01153 review final.docx [file pntd.0012446.s002.docx]

# Review of manuscript

“Effectiveness of ongoing single dose Rifampicin post-exposure prophylaxis (SDR PEP) implementation under routine programme conditions – an observational study in Nepal.
Short title: Impact assessment of single dose Rifampicin post-exposure prophylaxis.”

# Authors’ intentions

1. The title of this manuscript is: “Effectiveness of ongoing single dose Rifampicin post-exposure prophylaxis (SDR PEP) implementation under routine programme conditions – an observational study in Nepal
   Short title: Impact assessment of single dose Rifampicin post-exposure prophylaxis.”
2. The authors consider that doubts hinder the widespread implementation of SDR-PEP. They intend the findings to strengthen the evidence base for chemoprophylaxis with SDR-PEP.
3. The authors conducted a retrospective cohort study to compare “leprosy case notification rates and leprosy risk among close contacts”. They compared individuals and populations, respectively, in two districts implementing SDR-PEP (the intervention group) and two without (the control group).
4. Certain limitations of their approach are stated by the authors. Principally, they indicate that it was a retrospective observational study, not a randomized controlled trial (RCT).
5. The manuscript also reviews background literature.
6. Statistical procedures were applied to the data.
7. Findings and conclusions are stated, which will be reviewed in the light of what was done, against the background of relevant findings in the literature.

# Review

8. The manuscript describes an observational retrospective cohort study in four districts of Nepal, two of which were part of the earlier prospective LPEP study that had introduced single dose rifampicin post-exposure prophylaxis (SDR-PEP). The study protocol exists but it was not attached to the manuscript. The authors of this manuscript intended to evaluate the outcomes of adding SDR-PEP to contact screening, comparing these with outcomes in two other districts that are represented as having remained entirely without SDR-PEP but in most or all other respects not materially different from the intervention districts. The treated individuals resided in the LPEP districts and the comparator individuals resided in two other districts. The allocation of districts to LPEP or comparator groups was not randomized. Out of three LPEP districts one district named Parsa was non-randomly excluded from the study on the basis that from 2019 onwards the district effectively became a post-intervention district. Population-level case notification numbers over time in the four districts are also displayed in a figure.
9. The strength of the study is the insistence on using comparators, and the acknowledgement that confounders are important. Another strength is the forthright declaration by the authors of the intent behind the study, which is stated as being to strengthen the evidence base for chemoprophylaxis with SDR-PEP.
10. Unfortunately, in the respectful opinion of this reviewer, the authors’ findings and conclusions are erroneously attributed. Even the use of reasonably sophisticated statistical techniques and good field work cannot rescue the manuscript from the fundamental error or oversight. This reviewer apologizes in advance to the authors before continuing. None of what follows is intended to detract from respect for the enormous effort put into the field work or the statistical procedures carried out (although the Cox proportional hazards regression requires constant hazard ratio over time, which appears to be unavailable here from looking at the gap towards the right side in the Kaplan Meier graph, in Fig. 2. Also, Fig. 1 appears to deviate considerably from the numbers in Annex 1 for reasons that are not evident to this reviewer.)
11. In brief, the difference between comparator and intervention groups is fully explicable by the shorter average intervals between successive screenings of contacts in the comparator districts relative to the intervention districts. Previous studies in India from Jesudasan et al had shown that increasing the interval between screenings of household contacts from 1 year to 4 years produced roughly an 80% reduction in the detected incidence rate of clinical disease among the household contacts of lepromatous or borderline cases, and similarly roughly a 75% reduction in detected incidence rate among the household contacts of non-lepromatous types of cases. That apparent reduction of 75%+ was without any chemoprophylaxis at all.^[1]^
12. The authors here reduced the gap between screenings to as little as three months in the comparator districts, and then excluded the predictable huge boost in the detectable incidence rate of clinical disease in the comparator districts. This exclusion appears to have been arbitrary. The implication is that the field staff had missed these cases at first screening. In this reviewer’s respectful opinion, the evidence presented does not suggest suggest any error by the field staff. Rather, the authors might have overlooked the literature on rapid self-healing of some high-incidence types of clinical disease, between screenings.
13. Jesudasan et al showed that the incidence rate of detectable clinical disease drops by a factor of 4 or more (>75% of so-called “protection”) simply if the interval between screenings is prolonged from 1 year to 4 years, because this allows self-healing without sequelae in a large proportion of incident cases. If anyone is very keen to show 80% or 90% or 95% or even greater “protection”, they could simply reduce the inter-screening interval to 3 months or even less in the comparator group and simultaneously prolong the inter-screening interval to 7 years or even more in the intervention group. Any placebo that happens to accompany such prolongation of inter-survey interval in the intervention group relative to the comparator group will appear to offer “protection” against detectable clinical disease. With due respect to the authors, this is no “protection” at all. It is spurious because it is to a large extent, or even wholly, attributable to the difference in inter-screening interval between intervention and comparator groups. To drive home the point, the intervention could be any placebo substance or any action: eg., a vitamin tablet, immersion once in the Koshi River, one application of massage oil to exposed areas of skin, anything at all. As long as the interval between first and second screening is prolonged in the intervention group compared to the comparator group, even a completely ineffective placebo intervention will suffice to confer apparent “protection” from detectable clinical disease. The apparent association is spurious for the reasons explained above.
14. Accordingly, if they wish to proceed with a future manuscript using a similar approach then the authors might consider including the frequency of screening in each individual contact (number of screenings per unit time) as an independent variable, stratified by chronological year. This would be data in addition to the total person-years of observation contributed by that individual contact. The average duration of observation in the intervention districts is already reported to be longer than in comparator districts, consistent with a longer average screening interval in the intervention districts. In this reviewer’s opinion it would still be essential to make the frequency of screening in each individual explicit, and as precise as possible. For example, a study participant first examined on 1 July 2015 and then screened on 1 July 2016 and finally on 31 December 2023 without showing any signs of disease would have a frequency of screening during chronological year mid-2015 AD to mid 2016 AD calculated as 1 screening / 1 years = 1 screening per year for the chronological year mid-2015 to mid-2016. In each of the subsequent chronological years from 2 July 2016 to 31 December 2023 the frequency of screening would be 1 screening / 7.5 years = 0.13 screenings per year during each of the 7.5 chronological years starting mid-2016 and ending 31 December 2023. Another study participant first examined on 1 January 2023 and next screened on 1 July 2023 and found to have signs of disease would have a frequency of screening = 1 screening / 0.5 years = 2 screenings per year during the chronological year starting from mid-2022 AD (the Nepali BS calendar runs from approximately mid-year in the Gregorian calendar). The Kaplan Meier survival graph comparison between comparator and intervention groups would then be interpreted in strata (slices) of chronological years. This would help mitigate the confounding from the interval between screenings and from temporal trends in the differences between districts. Otherwise the findings and conclusions currently claimed by the authors, in the respectful opinion of this reviewer, would remain largely or wholly spurious.
15. Further, there appears to be a notable discrepancy between the numbers in Annex 1 and the lines drawn in Figure 1 of the manuscript. Annex 1 numbers when plotted look like this:

Index cases per million population per year

|  | Jhapa | Morang | Sunsari | Udayapur |
| --- | --- | --- | --- | --- |
| from mid 2015 | 233.747 | 203.969 | 138.814 | 94.9661 |
| from mid 2016 | 195.204 | 190.749 | 133.078 | 91.4488 |
| from mid 2017 | 172.824 | 177.528 | 110.134 | 137.173 |
| from mid 2018 | 343.161 | 142.589 | 96.3671 | 168.829 |
| from mid 2019 | 203.907 | 166.197 | 76.8642 | 123.104 |

It is notably different from Figure 1 in the manuscript. This reviewer is unable to explain the difference. Population effects need to be for the whole population, in this reviewer’s opinion, and not just for contacts. The discrepancy might have arisen from some unclearly disclosed or undisclosed exclusion of newly detected cases or contacts or index cases first screened in 2015 or later. The manuscript does not make entirely transparent what the authors actually did. This reviewer had to study the manuscript repeatedly and put together clues and hints to arrive at what the authors did. Even then, some perplexing features remain such as the discrepancy between Annex 1 and Figure 1 of the manuscript. Until a clear explanation is made available for the stated discrepancy, in this reviewer’s respectful opinion Fig. 1 of the manuscript remains invalid.

16. The authors have omitted the standard WHO-style monitoring information for these four study districts. Such monitoring information enables observations and trends to be placed in context and evaluated objectively by a reader. In particular, the WHO Weekly Epidemiological Record annual global updates include the following information year by year, at national level:
    16.1. No. of (resident) cases newly detected during that year
    16.2. No. of new MB cases
    16.3. No. of new cases with G2D
    16.4. No. of children in the population together with
          16.4.1. No. of new child cases
          16.4.2. No. of new child cases with G2D

16.5	No. of retreatment cases other than relapses

16.6	No. of relapses

16.7	No. of non-autochthonous cases

16.8	The above information for each of the four districts would be essential to place the study data in context, and for evaluation of differing delays in case detection between districts by reference to G2D% among newly detected cases. Additionally, for the benefit of readers unfamiliar with national epidemiological trends in Nepal, it would be desirable to include a visual depiction of trends, under the same headings, for the whole of Nepal as well as the four study districts.

17. There are other weaknesses in the manuscript. It does not appear necessary to go into them in great detail given the fundamental error in the manuscript. However, further discussion with a list of suggested improvements is included below out of respect for the efforts put in by the authors.

## Recommendation

18. This reviewer regretfully is constrained to recommend rejection of this manuscript based on the fundamental error of attribution as described above. However, the authors might consider taking on board suggestions for improvement including those listed above and below, to improve the manuscript to a point where the findings and conclusions are no longer largely or wholly spurious. Currently, in this reviewer’s respectful opinion, not only the findings and conclusions but even the title and the short title are not available on the evidence owing to the stated fundamental error of attribution.
19. For ethical reasons, in view of the fundamental error identified and the as yet unexplained discrepancy between Annex 1 and Figure 1 of the manuscript, the authors might consider how best to avoid misinformation. In particular, in this reviewer’s respectful opinion, the evidence in the manuscript does not in any way support the claim that “Integrating SDR-PEP within well-organized contact screening programs is effective and is expected to reduce leprosy transmission when applied as a rolling intervention.”

## For future reference

20. In any future manuscript, terminology needs to be defined precisely unless it is completely transparent and self-evident. eg., What exact definition of “blood relation” is used here? What is “index case”? What is the definition of “incident case” etc. What is the exact definition of “effectiveness” as used here, and what is the definition of “impact”? Impact is often understood to mean epidemiological impact at population level. Precise definitions would strengthen the validity of any claimed findings.
21. An important known confounder at the level of individual contacts is the number of missed or recrudescent lepromatous cases in that general area. Their nasal discharges can contain millions of viable bacilli per day even in the absence of prominent physical signs of disease.^[2]^ Lepromatous cases form a very small minority of all newly diagnosed cases but they have an overwhelmingly important role in supplying viable bacilli to others and to the environment. Differing frequencies of missed or recrudescent lepromatous cases in the four districts could explain an important part of the differences claimed to exist between intervention and comparator groups. The authors provide evidence in Table 2 that the comparator districts had more MB index cases, which include lepromatous cases. The number of viable bacilli per day in nasal discharges of even borderline lepromatous cases is scanty compared to lepromatous cases. Therefore, the confounder is not the total number or prevalence of all MB cases, but rather missed or recrudescent lepromatous cases in particular.
    Given the weight of this confounder, it would be essential in this reviewer’s opinion to include for each district:
    21.1.1. The number of skin or nasal smears taken at first or subsequent screenings
    21.1.2. The number of smears taken for which microscopy reports are available
    21.1.3. The number of positive smears among those taken
22. How many individuals who were ever close contacts of earlier index cases, even before first screening, and who were then first screened in 2015 or later, were excluded from the sampling frame of contacts?
    22.1. What were their characteristics in terms of known risk factors for detection of clinical disease, relative to close contacts included in the sampling frame in each district?
23. How is “case notification rate” defined by the authors? What exactly is the denominator of the rate as used in this manuscript, and over what duration of time (eg., 1 year)?
24. It would be essential in this reviewer’s opinion that the reader be provided with ready information for straightforward unadjusted estimates, alongside any statistical adjustments that the authors might choose to include. The use of the Kaplan Meier survival analysis is a strength of the manuscript, introducing a degree of transparency. In this reviewer’s opinion the time axis in the figure (Figure 2) is best labelled chronologically to assist readers (mid-2015 at extreme right, Dec 2023 at extreme left, if this reviewer has understood the figure correctly). Although this is not necessarily a criticism of the statistical approaches used, the data would also need to be tested for compatibility with the necessary assumptions (eg., for Cox proportional hazards regression) and the findings of such test reported alongside the output. Even if a constant hazard ratio over time is an unsafe assumption with these data, as is suggested by a quick glance at the conspicuous divergence between groups at the right in Figure 2 of the manuscript, there is nearly always yet another statistical approach that is appropriate and fit for purpose. In any case, alongside statistical adjustments, in this reviewer’s opinion it is essential to provide also the basic information for calculation of unadjusted Incidence Rate Ratio between the intervention and comparator groups. For example, in any future manuscript the information would include, district by district and chronological year by year:
    24.1. How many contacts entered the sampling frame by first examination during a stated year?
    24.2. How many of such contacts received SDR PEP?
    24.3. How many of such contacts from the sampling frame were randomly selected for the study in each district?
    24.4. How many person-years until clinical disease or censoring by loss to follow-up were contributed by these selected contacts?
    24.5. What was the incidence rate ratio of clinical disease between intervention and comparator groups during each chronological year given the number of person-years contributed by each contact during follow-up?
25. This is not an RCT. Intervention contacts lived in different districts from comparators. One district does not even share any border with the intervention districts. Therefore confounders are of great importance if the authors wish to make their findings valid. Should the authors be open to suggestions, then temporal trends in each district including Parsa before, during and after the introduction of SDR PEP in each district could help illuminate confounding arising from material differences between districts. As the manuscript stands, the non-random exclusion of Parsa district (one of only three LPEP districts in Nepal) is a major weakness in this reviewer’s opinion, given that the findings are meant to be generalized to the whole of the Terai districts, if not to the whole of Nepal.
    25.1. One district is seldom identical to another in all known and unknown confounding factors. For example, environmental and socio-economic factors, including sanitation or schooling level, did not remain constant in even Province 1 (Koshi Province) between 2015 and 2019 (source: Nepal National Planning Commission, Multidimensional Poverty Index 2021). Such transitions might have occurred at varying rates in different districts or even different municipalities of the province. Coming to other illustrative confounders, tree cover is known to vary between districts (source: Nepal Department of Forest Research and Survey, State of Nepal’s Forests 2015). A large river abuts only two of the districts. Therefore shade, moisture as well as exposure of surfaces to ultraviolet radiation are likely to vary between districts. Desikan and Sreevatsa^[3]^ demonstrated that M. leprae can remain viable for 5 months when dried and in the shade, or for 46 days in moist soil. Truman and Gillis^[4]^ demonstrated that UV radiation affects the viability of M. leprae. Such well-established confounders would need to be taken into account if the authors hope to demonstrate any persuasive associations by means of a retrospective cohort approach. Their claims might in due course differ markedly from the findings of the largest RCT that allowed a direct comparison between the rifampicin PEP (double dose) arm and a comparator arm without rifampicin PEP. Therefore it seems advisable to take all known measures to deal with confounding.
26. In this reviewer’s opinion, it is essential for ethical reasons to include a balanced 360 degree view in the background review. The authors’ background review fails to mention Cuba where SDR-PEP was introduced into routine services in 2002 with little or no impact on transmission over the decades, as evident from annual WHO global updates. Or Kiribati where after 2 years of intensive case-finding and contact screening from 2016, SDR-PEP for contacts was introduced from 2018 and used for 5 years (2018 to 2022), with an apparent increasing trend in new multibacillary cases among children.^[5]^ Or Alta Floresta in Mato Grosso, Brazil where the investigators reported “intense transmission”^[6]^ following the introduction of SDR-PEP for household contacts. A balanced 360 degree review would also include consideration of the best available outcomes from good case-finding, contact tracing and treatment without chemoprophylaxis, as demonstrated in several countries and districts showing relatively rapid and sustained declines in incidence rate of clinical disease. Ever since certain unfortunate events in the nineteen forties, those combating diseases are enjoined to go beyond merely advocating an intervention and to provide a 360 degree overview of all relevant information including known or potential risks of an intervention, so that policy makers and individuals can take adequately informed decisions.

1. Jesudasan K et al. Lepr Rev (1984) 55, 353-359
2. Davey TF, Rees RJ. Lepr Rev. 1974;45(2):121-34
3. Desikan & Sreevatsa. Lepr Rev 1995 Dec;66(4):287-95
4. Truman & Gillis. Int J Lepr Other Mycobact Dis 2000 Mar;68(1):11-7.
5. Campbell et al. Trop Med Infect Dis. 2024 Mar 1;9(3):58.
6. Machado et al. Infectious Diseases of Poverty (2022) 11:21 https://doi.org/10.1186/s40249-022-00943-7
