# Supplementary material for: Effectiveness of ongoing single dose rifampicin post-exposure prophylaxis (SDR-PEP) implementation under routine programme conditions—An observational study in Nepal
Source: PLoS Negl Trop Dis. 2024 Dec 4;18(12):e0012446. doi: 10.1371/journal.pntd.0012446 (PMC11649142; doi:10.1371/journal.pntd.0012446)
Supplement: Attachment — Submitted filename: PLOS NTD PNTD-D-24-01153 note to editor.docx [file pntd.0012446.s003.docx]

CONFIDENTIAL NOTE to PLOS NTD EDITOR(S)

Re: PLOS Neglected Tropical Diseases: Invitation to Review Manuscript (PNTD-D-24-01153)

| **Joel Almeida <leprosyinindia1@gmail.com>** | Mon, Aug 26, 4:44 PM |  |      |
| --- | --- | --- | --- |
| to PLOS  | | |  |

Dear Dr. Converse/Editor,

Greetings.

I accepted the invitation to review manuscript PNTD-D-24-01153.

With a view to transparency, I would like to put on record that I have written as follows in an email discussion group (LML) on 7 August 2024:

"Leprosy Mailing List –  August 7,  2024

 Ref.:  (LML)  Safety for the people of endemic countries
From: Joel Almeida, Mumbai, India

 Dear Pieter and colleagues,

Thanks to Wim van Brakel for his response (LML 3 Aug 2024).

He co-designed the LPEP study. He is the technical lead for NLR (Netherlands Leprosy Relief) who was the LPEP implementation partner for Dadra Nagar Haveli, India. What do Wim van Brakel's own co-authored data from 2015 to 2017 demonstrate? (1, 2 and attachment with key points encircled in red)

a) Pre-intervention baseline:
Zero newly diagnosed HD (leprosy) patients with G2D (Grade 2 disability)
Equivalent to less than 1 newly diagnosed HD patient with G2D per 374,000 population (<2.67 per million population/year)

b) During 9 quarters of administering PEP (post-exposure prophylaxis):
47 newly diagnosed HD patients with Grade 2 disability among 374,000 population
Equivalent to 56 newly diagnosed HD patients with G2D  per million population/year

56 / <2.67 = more than 21-fold boost in G2D following introduction of PEP

G2D or visible deformity usually indicates underlying permanent nerve damage. Affected limbs and eyes tend to deteriorate slowly but steadily,
from repeated painless wounds and infections
to attrition of extremities
to disintegration of extremities
and even to eventual amputation of extremities.

These are the consequences for the victims of PEP endorsed by Wim van Brakel, the Medical Director of NLR (Leprastichting).

Is it wrong to ensure safety for the families and other contacts of newly diagnosed HD patients, as required under various regulations and legislations? Are we opposed to respecting people in India, Brazil, Indonesia etc to the same extent as we respect people in Holland?

With all sincerity,

 Joel Almeida

PS Nearly all the most knowledgeable HD experts in the world are on LML. Is it wrong to put analyses before them for the widest expert scrutiny? Two or three reviewers might inadvertently allow serious errors to pass, such as the claim that PEP is safe. LML scrutiny is deeper, wider and therefore more reliable..

References

1.     Barth-Jaeggi T, Steinmann P, Mieras L, et al. Leprosy Post-ExposureProphylaxis (LPEP) programme: study protocol for evaluating the feasibility and impact on case detection rates of contact tracing and single dose rifampicin. BMJ Open 2016;6:e013633.doi:10.1136/bmjopen-2016-013633.

2.     Steinmann P, Cavaliero A, Aerts A et al. The Leprosy Post-Exposure Prophylaxis (LPEP) programme: update and interim analysis. Lepr Rev (2018) 89, 102–116"

That said, I believe that I can provide you with a fair, honest, and unbiased assessment of the strengths and weaknesses of the manuscript. If you judge otherwise given my disclosure above, would you mind letting me know please as soon as possible? Preferably within 24 hours if possible, because of the tight deadline for review.

With all sincerity,

Joel Almeida
