# Supplementary material for: Effectiveness of ongoing single dose rifampicin post-exposure prophylaxis (SDR-PEP) implementation under routine programme conditions—An observational study in Nepal
Source: PLoS Negl Trop Dis. 2024 Dec 4;18(12):e0012446. doi: 10.1371/journal.pntd.0012446 (PMC11649142; doi:10.1371/journal.pntd.0012446)
Supplement: Attachment — Submitted filename: Responses to the reviewers.docx [file pntd.0012446.s004.docx]

Nand Lal Banstola

Corresponding author

22 October 2024

Editorial Office

PLOS Neglected Tropical Diseases

Dear academic and section editors,

Thank you for the opportunity to revise and resubmit our manuscript, " Effectiveness of ongoing single dose Rifampicin post-exposure prophylaxis (SDR PEP) implementation under routine programme conditions—an observational study in Nepal" (Manuscript ID: PNTD-D-24-01153). We are grateful for the constructive feedback you and the reviewers provided, which has been invaluable in improving the quality and clarity of our work. Below, we offer a detailed response to each of the comments raised by the reviewers and outline the corresponding changes made in the revised manuscript.

**Responses to questions of Reviewer 1 and 2**

**Methods**

-Are the objectives of the study clearly articulated with a clear testable hypothesis stated?

-Is the study design appropriate to address the stated objectives?

-Is the population clearly described and appropriate for the hypothesis being tested?

-Is the sample size sufficient to ensure adequate power to address the hypothesis being tested?

-Were correct statistical analysis used to support conclusions?

-Are there concerns about ethical or regulatory requirements being met?

**Reviewer #1: This is a very well-conducted study of great interest. The report is well-written.**

**Response:** Thank you

**Reviewer #2: -	The sample size is sufficient to ensure adequate power to address authors' hypothesis**

-	However, some additional analysis could have been made:

o	Page 8, Figure 1: is the decrease in leprosy incidence significant? “the decline is most evident in the Morang and Jhapa district” sentence should be replaced by a sentence given the incidence rates and the comparison between those from the different districts.

**Response:** A sentence was added in the document, stating: The incidence rate per one million in Morang decreased from 343 in 2015 to 127 in 2021, and in Jhapa, it decreased from 216 in 2015 to 76.2 in 2021.

•	Table 2: p-value should be mentioned in order to easily identify the differences between the districts.

**Response:** We agree with the reviewer that the main purpose of Table 2 is to allow the reader to appreciate differences between the districts. However, given the large sample size, any observed difference will be statistically significant. Therefore we consider p-values less relevant here and prefer to just show absolute numbers and percentages.

•	Table 4: some p-values are 0.00 or 0.000, please clarify

**Response:** Thank you for pointing this out. To enhance clarity we have removed p-values where confidence intervals have been provided.

-	Page 6:

o	the dosage of RIF should be mentioned,

**Response:** Thank you for the suggestion, the dosages have been added

•	the possibility that people from one district travel in another should be discussed as this might interfere with the way to contract leprosy

**Response:** The contacts were selected based on the criteria described on page 7, among which: living in the same household or compound as the index case for at least three months, using the same kitchen and living under the same roof, taking into account that people may move, and contract an M. leprae infection or transmit the bacteria in another area than their area of origin.

-	Page 7: could the authors comment on the contact average par case that seems very high (i.e. 23) in comparison to literature and also specify the nature of their relationship, as it was shown previously that single-dose rifampicin offered significant protection for only neighbours of neighbours plus social contacts (reference 7).

**Response:** The number of contacts per index patient was higher than the standard 20 for contextual reasons. A mixed contact approach was used, targeting household contacts and neighbours, who are often relatives also. The average size of a household in Nepal is 5. Covering the index patient's household and neighbouring houses quickly adds up to more than 20.

- Single-dose rifampicin reduces the risk of developing leprosy in all close contacts. However, the risk reduction is bigger in neighbours than in family-related household contacts.

-	Page 10: regarding the diagnosis methods: the authors should clearly specify that the diagnosis was only based on clinic and provide some evidence that the abilities of the leprosy confirmation teams working in each district to diagnose leprosy were similar.

**Response:** This information is added: All team members received the same training, and their experience working in leprosy-endemic areas spanned at least eight years.

--------------------

Results

-Does the analysis presented match the analysis plan?

-Are the results clearly and completely presented?

-Are the figures (Tables, Images) of sufficient quality for clarity?

**Reviewer #1:** The results are clearly presented.

**Response:** Thank you.

**Reviewer #2: -**	Tables and figures:

•	it should be clearly indicated which districts represent the comparator and the intervention groups in all tables and figures

**Response:** Thank you, this information has been added.

•	there are many small tables, it should be possible to merge some of them (e.g. Tables 2 and 3, Tables 4 and 5)

**Response:**	Thank you for this comment. We have reorganized the tables and ensured that the format used in consistent.

-	Page 6: a map of Nepal highlighting the districts would be highly appreciated

**Response:** Thank you for the suggestion, a map has been included (Figure 1).

-	“discussion”:

o	page 16: the first sentences of the discussion should be replaced by more quantitative statements, the same can be said for the sentence “The individual protective effect of SDR-PEP is clear” page 19.

**Response:** page 16: the quantitative statements are made in the third sentence.

**Response:** page 19:

•	given the results section, is not straightforward to see where the “protective effect of 72%” come from

**Response:** This result comes from Table 6. In the text in the results section a reference has now been added to Table 6.

•	page 18: the authors should also comment on the risk of drug resistance selection in leprosy by given a single drug

**Response:** Thank you. A reference to this article Negligible risk of inducing resistance in Mycobacterium tuberculosis with single-dose rifampicin as post-exposure prophylaxis for leprosy - PubMed (nih.gov) has been included in the discussion section.

Conclusions

-Are the conclusions supported by the data presented?

-Are the limitations of analysis clearly described?

-Do the authors discuss how these data can be helpful to advance our understanding of the topic under study?

-Is public health relevance addressed?

**Reviewer #1:** The Conclusions are full supported by the data presented.

**Response:** Thank you

**Reviewer #2:** Please refer to the above sections

**Response:** See the responses above and the adaptations in the manuscript.

Editorial and Data Presentation Modifications? Use this section for editorial suggestions as well as relatively minor modifications of existing data that would enhance clarity. If the only modifications needed are minor and/or editorial, you may wish to recommend “Minor Revision” or “Accept”.

**Reviewer #1:** The Introduction is a very clear summary of current knowledge and practice in regard to post-exposure prophylaxis (PEP) for leprosy. I suggest adding a sentence about retrospective case-finding and PEP, which was done in Cambodia, with good results. This is probably a useful way to enhance the effect of PEP at the start of an intensive PEP programme. See: "Preventing leprosy with retrospective active case finding combined with single-dose rifampicin for contacts in a low endemic setting: results of the Leprosy Post-Exposure Prophylaxis program in Cambodia. Cavaliero, et al, Acta Tropica, 2021.

A second point relates to cases in children, which are regarded as a reasonable proxy for the level of transmission in a community. In Figure 1 of the current paper, the overall incidence rate of leprosy in each of the four districts is presented. If available, I would like to see the same Figure replicated to show the incidence in children under 15 years of age. Since there were only 123 incident cases in the study, the numbers may be too low to reach any conclusion, but it is an indicator that should be watched closely.

**Response:** Thank you for your suggestions.

The introduction now includes a sentence about retrospective case-finding and PEP, referencing the Cambodia publication.

**Response:** Figure 1 shows the overall trend of the new case detection rate in the districts (the numbers are not limited to the new patients detected in the study). However, as you suggested, a figure showing the new child case rates has been added (Figure 3).

**Reviewer #2:** Page 4: the authors should provide a table summarizing the design, number of cases and main findings of the PEP trials already published together with their one study

**Response:** Thank you for your suggestion. This information has been summarised in this paper: The State of Affairs in Post-Exposure Leprosy Prevention: A Descriptive Meta-Analysis on Immuno- and Chemo-Prophylaxis - PubMed (nih.gov)

A reference to this paper has been included in the introduction.

Summary and General Comments. Use this section to provide overall comments, discuss strengths/weaknesses of the study, novelty, significance, general execution and scholarship. You may also include additional comments for the author, including concerns about dual publication, research ethics, or publication ethics. If requesting major revision, please articulate the new experiments that are needed.

**Reviewer #1:** High quality study and paper.

**Response:** Thank you.

**Reviewer #2:** This study represents an enormous piece of work given the challenges of such clinical trials, especially among population whose socioeconomic status is predominantly low (poor and ultra-poor) and when the follow-up needs to be long as for leprosy. Therefore, I believe that after the modifications requested this paper deserves to be published, as it will add valuable data regarding PEP implementation in the leprosy field.

**Response:** Thank you.

# Review of manuscript – **reviewer 3**

“Effectiveness of ongoing single dose Rifampicin post-exposure prophylaxis (SDR PEP) implementation under routine programme conditions – an observational study in Nepal.
Short title: Impact assessment of single dose Rifampicin post-exposure prophylaxis.”

# Authors’ intentions

1. The title of this manuscript is: “Effectiveness of ongoing single dose Rifampicin post-exposure prophylaxis (SDR PEP) implementation under routine programme conditions – an observational study in Nepal
   Short title: Impact assessment of single dose Rifampicin post-exposure prophylaxis.”
2. The authors consider that doubts hinder the widespread implementation of SDR-PEP. They intend the findings to strengthen the evidence base for chemoprophylaxis with SDR-PEP.
3. The authors conducted a retrospective cohort study to compare “leprosy case notification rates and leprosy risk among close contacts”. They compared individuals and populations, respectively, in two districts implementing SDR-PEP (the intervention group) and two without (the control group).
4. Certain limitations of their approach are stated by the authors. Principally, they indicate that it was a retrospective observational study, not a randomized controlled trial (RCT).
5. The manuscript also reviews background literature.
6. Statistical procedures were applied to the data.
7. Findings and conclusions are stated, which will be reviewed in the light of what was done, against the background of relevant findings in the literature.

# Review

8. The manuscript describes an observational retrospective cohort study in four districts of Nepal, two of which were part of the earlier prospective LPEP study that had introduced single dose rifampicin post-exposure prophylaxis (SDR-PEP). The study protocol exists but it was not attached to the manuscript. The authors of this manuscript intended to evaluate the outcomes of adding SDR-PEP to contact screening, comparing these with outcomes in two other districts that are represented as having remained entirely without SDR-PEP but in most or all other respects not materially different from the intervention districts. The treated individuals resided in the LPEP districts and the comparator individuals resided in two other districts. The allocation of districts to LPEP or comparator groups was not randomized. Out of three LPEP districts one district named Parsa was non-randomly excluded from the study on the basis that from 2019 onwards the district effectively became a post-intervention district. Population-level case notification numbers over time in the four districts are also displayed in a figure.
9. The strength of the study is the insistence on using comparators, and the acknowledgement that confounders are important. Another strength is the forthright declaration by the authors of the intent behind the study, which is stated as being to strengthen the evidence base for chemoprophylaxis with SDR-PEP.
10. Unfortunately, in the respectful opinion of this reviewer, the authors’ findings and conclusions are erroneously attributed. Even the use of reasonably sophisticated statistical techniques and good field work cannot rescue the manuscript from the fundamental error or oversight. This reviewer apologizes in advance to the authors before continuing. None of what follows is intended to detract from respect for the enormous effort put into the field work or the statistical procedures carried out (although the Cox proportional hazards regression requires constant hazard ratio over time, which appears to be unavailable here from looking at the gap towards the right side in the Kaplan Meier graph, in Fig. 2. Also, Fig. 1 appears to deviate considerably from the numbers in Annex 1 for reasons that are not evident to this reviewer.)

**Response:** The table in Annex 1 presents the number of index cases whose contacts were screened and the number of contacts screened per year per district in 2015-2019. During LPEP, prospective index patients were included, indeed, from 2015 to 2019, as well as retrospective index cases that were diagnosed in the years 2012 to 2014. This means some index cases included in LPEP, particularly in 2015, were diagnosed in previous years. Fig. 1 presents rates, including the total number of reported new cases per 1 million population per year in each district. Fig. 1 is based on data routinely collected from the leprosy control programmes in these districts. Annex 1 presents the number of patients of which contacts were screened and the number of contacts screened per year and Fig.1 presents the routinely collected data.

For the Cox regression and the Kaplan-Meier curve we did test the proportional hazards assumption and obtained a p-value of 0.75, so no indication that the proportional hazards assumption was not met. The reason for the somewhat steeper drop at the right end of the curve in comparator districts is that in those districts there were very few observations left at that point. As indicated in Table 3, average follow-up time was shorter in comparator districts. We added the following to the results section: ‘The test for the proportional hazards assumption resulted in a chi square of 0.10, *p*=0.75, indicating that the assumption was met’.

11. In brief, the difference between comparator and intervention groups is fully explicable by the shorter average intervals between successive screenings of contacts in the comparator districts relative to the intervention districts. Previous studies in India from Jesudasan et al had shown that increasing the interval between screenings of household contacts from 1 year to 4 years produced roughly an 80% reduction in the detected incidence rate of clinical disease among the household contacts of lepromatous or borderline cases, and similarly roughly a 75% reduction in detected incidence rate among the household contacts of non-lepromatous types of cases. That apparent reduction of 75%+ was without any chemoprophylaxis at all.^[1]^

**Response:** We agree with the reviewer that if successive screenings are carried out with long intervals in between, the yield of active screening will be lower than when intervals are shorter. However in this study there were no successive screenings, screenings were performed among different sets of contacts, triggered by identification of new index cases. It is true that in the intervention districts average follow-up time was longer than in the comparator districts (~82 months vs. ~64 months) but this is taken into account in the Cox regression model which uses total follow-up time as denominator. As can be seen from the Kaplan-Meier graph, intervention and comparator districts start to diverge from one another almost right from the start, there is no differential effect over time (also see previous comment on proportional hazards assumption).

12. The authors here reduced the gap between screenings to as little as three months in the comparator districts, and then excluded the predictable huge boost in the detectable incidence rate of clinical disease in the comparator districts. This exclusion appears to have been arbitrary. The implication is that the field staff had missed these cases at first screening. In this reviewer’s respectful opinion, the evidence presented does not suggest any error by the field staff. Rather, the authors might have overlooked the literature on rapid self-healing of some high-incidence types of clinical disease, between screenings.

**Response:** As explained above, there were no serial screenings. There was a first screening whenever an index case was identified and a final screening between Juny 2022 and December 2023. We excluded cases identified within three months of initial screening because of potential reporting delays. We wanted to avoid the possibility that a case detected at time of initial screening would thus be reported as an incident case, rather than a prevalent case at baseline. The 17 patients thus excluded were all from comparator districts. Not excluding them would have made the protective effect of SDR-PEP appear even stronger.

13. Jesudasan et al showed that the incidence rate of detectable clinical disease drops by a factor of 4 or more (>75% of so-called “protection”) simply if the interval between screenings is prolonged from 1 year to 4 years, because this allows self-healing without sequelae in a large proportion of incident cases. If anyone is very keen to show 80% or 90% or 95% or even greater “protection”, they could simply reduce the inter-screening interval to 3 months or even less in the comparator group and simultaneously prolong the inter-screening interval to 7 years or even more in the intervention group. Any placebo that happens to accompany such prolongation of inter-survey interval in the intervention group relative to the comparator group will appear to offer “protection” against detectable clinical disease. With due respect to the authors, this is no “protection” at all. It is spurious because it is to a large extent, or even wholly, attributable to the difference in inter-screening interval between intervention and comparator groups. To drive home the point, the intervention could be any placebo substance or any action: eg., a vitamin tablet, immersion once in the Koshi River, one application of massage oil to exposed areas of skin, anything at all. As long as the interval between first and second screening is prolonged in the intervention group compared to the comparator group, even a completely ineffective placebo intervention will suffice to confer apparent “protection” from detectable clinical disease. The apparent association is spurious for the reasons explained above.

**Response:** We thank the reviewer for pointing this out and agree that extending the interval between initial and final screening could increase the probability of self-healing and therefore reduce reported incidence rates. Jesudasan et al. state that: ‘when the between-survey interval increased from 1 to 3 years, the estimated incidence rate of leprosy was halved.’ However, as shown in Table 1 of the same article, this effect is not linear, most of the decrease is observed in the first years of additional follow-up. When the difference increases from 4 to 5 years, the effect on incidence rate is minimal, 1.19 versus 1.16 per 1000 person years at risk. In our study, subjects were screened with on average 5.5-year gaps in the comparator districts versus 7-year gaps in the intervention districts. It is therefore highly unlikely that differences in intervals between screening and associated differences in self-healing can explain the strong effect observed (72% reduction). We have, however, added to the limitations the following statement: ‘In addition average follow-up time was shorter in comparator districts than in intervention districts, ~5½ vs. ~7 years, which may have resulted in more self-healing in the intervention districts’, with reference to the article cited.

14. Accordingly, if they wish to proceed with a future manuscript using a similar approach then the authors might consider including the frequency of screening in each individual contact (number of screenings per unit time) as an independent variable, stratified by chronological year.

**Response:** Contacts were only screened twice, unless they were a contact again of a new index case. Once at baseline, and, for this study, a random sample was re-screened three years after the LPEP project ended.

This would be data in addition to the total person-years of observation contributed by that individual contact. The average duration of observation in the intervention districts is already reported to be longer than in comparator districts, consistent with a longer average screening interval in the intervention districts. In this reviewer’s opinion it would still be essential to make the frequency of screening in each individual explicit, and as precise as possible.

For example, a study participant first examined on 1 July 2015 and then screened on 1 July 2016 and finally on 31 December 2023 without showing any signs of disease would have a frequency of screening during chronological year mid-2015 AD to mid 2016 AD calculated as 1 screening / 1 years = 1 screening per year for the chronological year mid-2015 to mid-2016. In each of the subsequent chronological years from 2 July 2016 to 31 December 2023 the frequency of screening would be 1 screening / 7.5 years = 0.13 screenings per year during each of the 7.5 chronological years starting mid-2016 and ending 31 December 2023. Another study participant first examined on 1 January 2023 and next screened on 1 July 2023 and found to have signs of disease would have a frequency of screening = 1 screening / 0.5 years = 2 screenings per year during the chronological year starting from mid-2022 AD (the Nepali BS calendar runs from approximately mid-year in the Gregorian calendar).

**Response:** What the reviewer describes is not what has happened in this study. Contacts were not re-screened repeatedly. A random sample of contacts was re-screened to look at the effectiveness of ongoing SDR-PEP implementation under routine programme conditions.

This was done in 2022/2023 in both intervention and control areas. If there is a section in the manuscript that suggests repeated contact screenings in the same individual, we would be happy to revise that, but we are not sure on what section the reviewer bases this understanding.

The Kaplan Meier survival graph comparison between comparator and intervention groups would then be interpreted in strata (slices) of chronological years. This would help mitigate the confounding from the interval between screenings and from temporal trends in the differences between districts. Otherwise the findings and conclusions currently claimed by the authors, in the respectful opinion of this reviewer, would remain largely or wholly spurious.

**Response**: As the Kaplan-Meier curve clearly shows, the proportional hazards assumption was not violated. Only at the right tail of the curve, when few subjects were left in the comparator cohort, there is some diversion. The fact that the protective effect did not vary significantly with interval between baseline and final screening was also shown by the statistical test of the proportional hazards assumption which gave a p-value of 0.75. We added the following to the results section: ‘The test for the proportional hazards assumption resulted in a chi square of 0.10, *p*=0.75, indicating that the assumption was met’.

15. Further, there appears to be a notable discrepancy between the numbers in Annex 1 and the lines drawn in Figure 1 of the manuscript. Annex 1 numbers when plotted look like this:

Index cases per million population per year

|  | Jhapa | Morang | Sunsari | Udayapur |
| --- | --- | --- | --- | --- |
| from mid 2015 | 233.747 | 203.969 | 138.814 | 94.9661 |
| from mid 2016 | 195.204 | 190.749 | 133.078 | 91.4488 |
| from mid 2017 | 172.824 | 177.528 | 110.134 | 137.173 |
| from mid 2018 | 343.161 | 142.589 | 96.3671 | 168.829 |
| from mid 2019 | 203.907 | 166.197 | 76.8642 | 123.104 |

It is notably different from Figure 1 in the manuscript. This reviewer is unable to explain the difference. Population effects need to be for the whole population, in this reviewer’s opinion, and not just for contacts. The discrepancy might have arisen from some unclearly disclosed or undisclosed exclusion of newly detected cases or contacts or index cases first screened in 2015 or later. The manuscript does not make entirely transparent what the authors actually did. This reviewer had to study the manuscript repeatedly and put together clues and hints to arrive at what the authors did. Even then, some perplexing features remain such as the discrepancy between Annex 1 and Figure 1 of the manuscript. Until a clear explanation is made available for the stated discrepancy, in this reviewer’s respectful opinion Fig. 1 of the manuscript remains invalid.

**Response:** See answer to question number 10.

16. The authors have omitted the standard WHO-style monitoring information for these four study districts. Such monitoring information enables observations and trends to be placed in context and evaluated objectively by a reader. In particular, the WHO Weekly Epidemiological Record annual global updates include the following information year by year, at national level:
    16.1. No. of (resident) cases newly detected during that year
    16.2. No. of new MB cases
    16.3. No. of new cases with G2D
    16.4. No. of children in the population together with
          16.4.1. No. of new child cases
          16.4.2. No. of new child cases with G2D

16.5	No. of retreatment cases other than relapses

16.6	No. of relapses

16.7	No. of non-autochthonous cases

16.8	The above information for each of the four districts would be essential to place the study data in context, and for evaluation of differing delays in case detection between districts by reference to G2D% among newly detected cases. Additionally, for the benefit of readers unfamiliar with national epidemiological trends in Nepal, it would be desirable to include a visual depiction of trends, under the same headings, for the whole of Nepal as well as the four study districts.

**Response:** We added additional child rate information in Fig. 3 The other indicators, while interesting, are not directly relevant to the topic of this paper. Information about new MB cases in the cohorts is already included.

17. There are other weaknesses in the manuscript. It does not appear necessary to go into them in great detail given the fundamental error in the manuscript. However, further discussion with a list of suggested improvements is included below out of respect for the efforts put in by the authors.

**Response:** We thank the reviewer for providing these suggestions for improvements and hope our responses to his earlier comments have taken away most of his concerns.

## Recommendation

18. This reviewer regretfully is constrained to recommend rejection of this manuscript based on the fundamental error of attribution as described above. However, the authors might consider taking on board suggestions for improvement including those listed above and below, to improve the manuscript to a point where the findings and conclusions are no longer largely or wholly spurious. Currently, in this reviewer’s respectful opinion, not only the findings and conclusions but even the title and the short title are not available on the evidence owing to the stated fundamental error of attribution.

**Response:** With the responses above, we hope to have clarified that there is no fundamental error. We will continue to go through the recommendations below.

19. For ethical reasons, in view of the fundamental error identified and the as yet unexplained discrepancy between Annex 1 and Figure 1 of the manuscript, the authors might consider how best to avoid misinformation. In particular, in this reviewer’s respectful opinion, the evidence in the manuscript does not in any way support the claim that “Integrating SDR-PEP within well-organized contact screening programs is effective and is expected to reduce leprosy transmission when applied as a rolling intervention.”

**Response:** These points were addressed in the section above. The authors wish to respectfully disagree with the reviewer.

## For future reference

20. In any future manuscript, terminology needs to be defined precisely unless it is completely transparent and self-evident. eg., What exact definition of “blood relation” is used here? What is “index case”? What is the definition of “incident case” etc. What is the exact definition of “effectiveness” as used here, and what is the definition of “impact”? Impact is often understood to mean epidemiological impact at population level. Precise definitions would strengthen the validity of any claimed findings.

**Response:** The definition of the terminology used follows the WHO Guidelines on Leprosy Diagnosis, Treatment and Prevention and the WHO Technical Guidance on Leprosy/Hansen disease: contact tracing and post-exposure prophylaxis, as is apparent from the referencing.

The effectiveness is defined primarily as a reduced incidence among contacts screened in the intervention area compared to the control area. This is described in the introduction text as well as in the methodology section.

21. An important known confounder at the level of individual contacts is the number of missed or recrudescent lepromatous cases in that general area. Their nasal discharges can contain millions of viable bacilli per day even in the absence of prominent physical signs of disease.^[2]^ Lepromatous cases form a very small minority of all newly diagnosed cases but they have an overwhelmingly important role in supplying viable bacilli to others and to the environment. Differing frequencies of missed or recrudescent lepromatous cases in the four districts could explain an important part of the differences claimed to exist between intervention and comparator groups. The authors provide evidence in Table 2 that the comparator districts had more MB index cases, which include lepromatous cases. The number of viable bacilli per day in nasal discharges of even borderline lepromatous cases is scanty compared to lepromatous cases. Therefore, the confounder is not the total number or prevalence of all MB cases, but rather missed or recrudescent lepromatous cases in particular.
    Given the weight of this confounder, it would be essential in this reviewer’s opinion to include for each district:
    21.1.1. The number of skin or nasal smears taken at first or subsequent screenings
    21.1.2. The number of smears taken for which microscopy reports are available
    21.1.3. The number of positive smears among those taken

**Response**: We acknowledge that the proportion of MB index cases was higher in the comparator districts but when we assessed potential confounding we concluded that the classification of the index case did not have much impact on the estimate of the protective effect of PEP. When including MB index case in the model the HR changed from 0.35 to 0.37, less than the predefined threshold of 10% mentioned in the methods section.

22. How many individuals who were ever close contacts of earlier index cases, even before first screening, and who were then first screened in 2015 or later, were excluded from the sampling frame of contacts?

**Response:** The screening of contacts in 2022/2023 was done on a random sample of all screened contacts in the 2015-2019 period.

22.1. What were their characteristics in terms of known risk factors for detection of clinical disease, relative to close contacts included in the sampling frame in each district?

**Response:** See answer to previous questions.

23. How is “case notification rate” defined by the authors? What exactly is the denominator of the rate as used in this manuscript, and over what duration of time (eg., 1 year)?

**Response:** The case notification rate is the number of new patients reported to have leprosy. It is similar to the case detection rate, with a subtle difference being the fact that the notified cases might slightly differ from the actual number of detected cases. We work with the numbers reported, that is why we use the ‘case notification rate’. It is a term regularly used in Public Health and epidemiology.

24. It would be essential in this reviewer’s opinion that the reader be provided with ready information for straightforward unadjusted estimates, alongside any statistical adjustments that the authors might choose to include. The use of the Kaplan Meier survival analysis is a strength of the manuscript, introducing a degree of transparency. In this reviewer’s opinion the time axis in the figure (Figure 2) is best labelled chronologically to assist readers (mid-2015 at extreme right, Dec 2023 at extreme left, if this reviewer has understood the figure correctly). Although this is not necessarily a criticism of the statistical approaches used, the data would also need to be tested for compatibility with the necessary assumptions (eg., for Cox proportional hazards regression – this was done, see page 10 in the manuscript) and the findings of such test reported alongside the output. Even if a constant hazard ratio over time is an unsafe assumption with these data, as is suggested by a quick glance at the conspicuous divergence between groups at the right in Figure 2 of the manuscript, there is nearly always yet another statistical approach that is appropriate and fit for purpose. In any case, alongside statistical adjustments, in this reviewer’s opinion it is essential to provide also the basic information for calculation of unadjusted Incidence Rate Ratio between the intervention and comparator groups. For example, in any future manuscript the information would include, district by district and chronological year by year:
    24.1. How many contacts entered the sampling frame by first examination during a stated year?
    24.2. How many of such contacts received SDR PEP?
    24.3. How many of such contacts from the sampling frame were randomly selected for the study in each district?
    24.4. How many person-years until clinical disease or censoring by loss to follow-up were contributed by these selected contacts?
    24.5. What was the incidence rate ratio of clinical disease between intervention and comparator groups during each chronological year given the number of person-years contributed by each contact during follow-up?

**Response:** Participants entered the study at different points in time, depending on when an index case was reported. Each time an index case was reported, contacts were screened and provided PEP if they were living in an intervention district and not themselves diagnosed with leprosy at time of first screening. In comparator districts, contacts were also screened but no PEP was provided. Implementation of this contact screening strategy started earlier in the intervention districts, hence the longer average follow-up time (82 months vs. 64 months). Those enrolled in the study were screened a second time between July 2022 and December 2023. For this reason on the X-axis we show follow-up time rather than calendar time. As mentioned earlier, we performed a test for the proportional hazards assumption, which showed a p-value of 0.75. This was added to the results section.

25. This is not an RCT. Intervention contacts lived in different districts from comparators. One district does not even share any border with the intervention districts. Therefore confounders are of great importance if the authors wish to make their findings valid. Should the authors be open to suggestions, then temporal trends in each district including Parsa before, during and after the introduction of SDR PEP in each district could help illuminate confounding arising from material differences between districts. As the manuscript stands, the non-random exclusion of Parsa district (one of only three LPEP districts in Nepal) is a major weakness in this reviewer’s opinion, given that the findings are meant to be generalized to the whole of the Terai districts, if not to the whole of Nepal.

**Response:** The implementation of SDR-PEP was not continued as part of the routine programme in Parsa, therefore it was not included in this study that was aimed at comparing routine contact screening + SDR-PEP administration to contact screening alone, as is explained in the methodology section.

25.1. One district is seldom identical to another in all known and unknown confounding factors. For example, environmental and socio-economic factors, including sanitation or schooling level, did not remain constant in even Province 1 (Koshi Province) between 2015 and 2019 (source: Nepal National Planning Commission, Multidimensional Poverty Index 2021). Such transitions might have occurred at varying rates in different districts or even different municipalities of the province. Coming to other illustrative confounders, tree cover is known to vary between districts (source: Nepal Department of Forest Research and Survey, State of Nepal’s Forests 2015). A large river abuts only two of the districts. Therefore shade, moisture as well as exposure of surfaces to ultraviolet radiation are likely to vary between districts. Desikan and Sreevatsa^[3]^ demonstrated that M. leprae can remain viable for 5 months when dried and in the shade, or for 46 days in moist soil. Truman and Gillis^[4]^ demonstrated that UV radiation affects the viability of M. leprae. Such well-established confounders would need to be taken into account if the authors hope to demonstrate any persuasive associations by means of a retrospective cohort approach. Their claims might in due course differ markedly from the findings of the largest RCT that allowed a direct comparison between the rifampicin PEP (double dose) arm and a comparator arm without rifampicin PEP. Therefore it seems advisable to take all known measures to deal with confounding.

**Response:** Thank you for these interesting examples. By selecting two distinct districts as intervention areas and two comparable districts as control areas, all within the same province and with similar geographical and population characteristics, we have taken steps to minimize confounding as much as possible. In addition, we have measured and included known confounders in the multivariate analysis.

26. In this reviewer’s opinion, it is essential for ethical reasons to include a balanced 360 degree view in the background review. The authors’ background review fails to mention Cuba where SDR-PEP was introduced into routine services in 2002 with little or no impact on transmission over the decades, as evident from annual WHO global updates. Or Kiribati where after 2 years of intensive case-finding and contact screening from 2016, SDR-PEP for contacts was introduced from 2018 and used for 5 years (2018 to 2022), with an apparent increasing trend in new multibacillary cases among children.^[5]^ Or Alta Floresta in Mato Grosso, Brazil where the investigators reported “intense transmission”^[6]^ following the introduction of SDR-PEP for household contacts. A balanced 360 degree review would also include consideration of the best available outcomes from good case-finding, contact tracing and treatment without chemoprophylaxis, as demonstrated in several countries and districts showing relatively rapid and sustained declines in incidence rate of clinical disease. Ever since certain unfortunate events in the nineteen forties, those combating diseases are enjoined to go beyond merely advocating an intervention and to provide a 360 degree overview of all relevant information including known or potential risks of an intervention, so that policy makers and individuals can take adequately informed decisions.

**Response**: In the introduction section (Page 4) we have provided an overview of relevant studies. We can only refer to published research results. Please note that the article of Machado *et al.* from Brazil quoted by the reviewer does not conclude that intense transmission is the result of SDR-PEP implementation, it only states that such intense transmission was present in the areas selected for the study. It even advocates the use of SDR-PEP for successful interruption of *M. leprae* transmission. We have inserted additional references to the most recent PEP studies, such as the PEOPLE project.

We hope that these answers and revisions satisfactorily address the reviewers' and editor’s concerns. We thank you once again for considering our manuscript for publication in PLOS Neglected Tropical Diseases.

Sincerely,

Nand Lal Banstola

1. Jesudasan K et al. Lepr Rev (1984) 55, 353-359
2. Davey TF, Rees RJ. Lepr Rev. 1974;45(2):121-34
3. Desikan & Sreevatsa. Lepr Rev 1995 Dec;66(4):287-95
4. Truman & Gillis. Int J Lepr Other Mycobact Dis 2000 Mar;68(1):11-7.
5. Campbell et al. Trop Med Infect Dis. 2024 Mar 1;9(3):58.
6. Machado et al. Infectious Diseases of Poverty (2022) 11:21 https://doi.org/10.1186/s40249-022-00943-7
